# The Practicality of Post-mortem Imaging in Prenatal, Perinatal, and Pediatric Cases

**DOI:** 10.7759/cureus.28859

**Published:** 2022-09-06

**Authors:** Christina Ashby, Abrahim N Razzak, Ann Kogler, Ahmad Amireh, John Dempsey, Keldon K Lin, Joseph Waller, Pinky Jha

**Affiliations:** 1 Contract Research Organization, IQVIA Holdings, Inc., Durham, USA; 2 School of Medicine, Medical College of Wisconsin, Milwaukee, USA; 3 College of Medicine, Drexel University, Philadelphia, USA; 4 Movement Science and Education, Columbia University, New York City, USA; 5 College of Medicine, Boston College, Chestnut Hill, USA; 6 School of Medicine, Mayo Clinic, Scottsdale, USA; 7 Internal Medicine, Medical College of Wisconsin, Milwaukee, USA

**Keywords:** pediatric radiology, imaging modalities, pediatrics medicine, post-mortem computed tomography (pmct), post-mortem

## Abstract

The essential role of the autopsy is seen in its contributions to medical care, scientific research, and family counseling. Major contributions are also noted in forensic pathology as a means to determine cause-of-death for legal and medical experts. However, autopsy acceptance rates are quite low due to an array of reasons including delayed burials, faith, and moral burdening. Thus, non-invasive post-mortem imaging strategies are becoming increasingly popular. The objective of this literature review is to evaluate the strengths and weaknesses of numerous post-mortem imaging modalities and consider their benefits over the traditional autopsy. The need for expertise in image interpretation for pediatric and perinatal cases is also discussed. A variety of publications, totaling 32 pieces, were selected from available literature on the basis of relevance. These articles studied various perinatal and pediatric post-mortem imaging strategies and their applications in clinical practice. Key strategies include post-mortem MRI, post-mortem CT, fetal post-mortem sonography, post-mortem computed tomographic angiography, and three-dimensional surface scanning. There is a general consensus that no standard model for post-mortem imaging currently exists in the United States and European countries. Amongst the imaging modems studied, post-mortem MRI has been acknowledged to show the greatest promise in diagnostic accuracy for fetal age groups. Most studies demonstrated that post-mortem CT had limited use for autopsy. Post-mortem imaging strategies for autopsy have high potential given their minimal invasiveness and increasing popularity. Furthermore, it is vital to crafting a global standard procedure for post-mortem imaging for prenatal, perinatal, and pediatric cases to better understand the cause of death, decomposition factors, and effects in-utero, and to provide an alternative to traditional autopsy.

## Introduction and background

Autopsies in prenatal, perinatal, and pediatric cases provide useful information that enables epidemiologists to lessen or prevent instances of abnormality and illness while in utero. Additionally, autopsies may help obstetric and gynecologist physicians better advise their patients regarding future pregnancies and identify potential cases of abuse. Understanding the intricacies surrounding the cause of death in these vulnerable populations is essential. The only question that remains is: what is the optimal way to achieve this? A traditional autopsy is incredibly invasive and, in fetal and pediatric cases, has low acceptance rates in the United States (12%) and the United Kingdom (15%) [1]. Already grieving parents cite moral grounds, religious beliefs, and fear of disfigurement as the leading causes of hesitancy [2]. Yet, if the bodies are never examined post-mortem, crucial information that may be gleaned will irrevocably be buried, leaving the medical community unable to help future parents and children avoid the same fate. As a result, post-mortem imaging (PMI) is becoming increasingly popular although traditional autopsy may continue to be the method of choice for clinicians [3,4]. Non-invasive methods such as post-mortem computed tomography (PMCT), post-mortem magnetic resonance imaging (PMMRI), fetal post-mortem sonography, and other imaging modems allow for the advancement of the scientific community behind post-mortem diagnostics while respecting the beliefs and sentiments of the family. After all, these imaging modalities are less of an invasive option compared to the traditional autopsy. In this literature review, the benefits of PMI for prenatal, perinatal, and pediatric cases in conjunction with traditional autopsy will be discussed, as well as various methods of imaging, the problems each pose, and the intricacies involved in interpreting imaging in various stages of fetal and pediatric development.

Methods

The PubMed database was searched for pertinent publications made available in the last 10 years (2010-2020). Search terms included post-mortem MRI, post-mortem CT, fetal post-mortem sonography, post-mortem computed tomographic angiography (PMCTA), and three-dimensional surface scanning (3DSS). Articles with redundant information, overlapping patient cohorts, and preclinical studies were excluded. Studies included relevant information regarding the methodology of PMI in various countries, intricacies in taking and interpreting scans from developing fetuses, and various types of imaging. Ultimately, 32 publications were selected from the literature. These articles studied various perinatal and pediatric PMI strategies and their applications in clinical practice.

## Review

Benefits of traditional autopsy

Traditional autopsy allows for the diagnosis of conditions that may have been missed in life. It has the capability to help construct pathological profiles of diseases, provide justification in ruling causes of death, highlight conditions to surviving family members, and provide invaluable training and education to emerging physicians and researchers [4]. Failing to conduct an autopsy stagnates medical advancement. Despite this, an autopsy is utilized in only 10% of all deaths in the United States [4]. Autopsy rates in infant and neonatal cases specifically have shown to be around 30% lower when compared to worldwide autopsy rates [5,6].

Motivation for PMI

Though the subject matter may seem grim, pediatric PMI is an essential tool in understanding and performing diagnostic evaluation for the cause of death in the perinatal, neonatal, and pediatric patient populations. Perinatal mortality rate refers to infant deaths of more than 24 weeks of gestation but less than seven days of age per 1,000 live births [5]. Conducting autopsies is important, as studies have shown a 10%-25% discrepancy between the perceived cause of death and findings from traditional autopsies [5]. In fact, up to half of all medical certificates for stillborn deaths might be incorrect due to this inconsistency [5]. Despite the necessity of this imaging, barriers remain.

Autopsies are oftentimes rejected on the basis of religious beliefs, fear of unethical practices, high cost, and the fact that the next of kin believes the deceased should be allowed to rest in peace along with an emphasis on individual choices [7]. In 80%-90% of cases, clinicians discuss autopsy options and parents decline due to moral or religious grounds, delay of funerals, or disfigurements [2]. PMI has therefore been used as a precise alternative measuring tool both as a standalone method and in conjunction with traditional autopsy. Creating a connection for emerging scientists and physicians in the field to give a consensus on PMI while recognizing existing and potential barriers may contribute to eventually overcoming these barriers [7].

At the same time, it is important to analyze the challenges we have ahead when considering the newly built PMI apparatus. Because fewer people opt for fetal and pediatric autopsy, this stunts information that could be used to advise parents regarding future pregnancies or contribute to epidemiological studies. A study conducted by the European Society of Pediatric Radiology concluded that there was both no consensus regarding which sub-population of post-mortem cases to image- with the options being between fetal, neonatal, stillbirths, or infant deaths - and that less than one-third of centers in Europe have standardized protocols for PMI [1].

Health professionals and coroners viewed less invasive autopsy as a positive development in perinatal and pediatric care [3]. However, there needs to be wider access to such perinatal and pediatric imaging systems for patients and medical conventions to account for those who oppose conventional autopsy. Forensic practitioner training regarding the intricacies of fetal development and post-mortem anatomical changes is also required to accurately record data [8-10].

PMI techniques

PMI is not a one-size-fits-all model - different methods will better enable examiners to uncover traumas that others may otherwise overlook. Post-mortem radiology, fetal post-mortem sonography, PMCT, PMCTA, PMMRI, and 3DSS are some of the primary modalities of PMI.

Post-mortem Radiology

Post-mortem radiology is the most common PMI service provided in Europe, with 80% of surveyed practitioners reporting the use of this method [1]. This term refers to conventional radiography imaging systems that have been classically used in radiology services, oftentimes x-rays to diagnose, and assess any bony abnormalities and ossification centers depending on the fetal gestational age. Post-mortem fetal radiography is integral to the accurate diagnosis of skeletal dysplasia which could have manifested in the fetus [1]. Interpreting such results will require training for diagnosticians to understand different gestational ages in addition to the appearance of common dysplasia [1].

Diagnosing and assessing such bony abnormalities after a neonatal or pediatric death is difficult: the positional changes this method necessitates are not possible due to rigor mortis [11]. There is no standardized protocol for this, and PMI in all cases has a low diagnostic yield in clinical practice, rendering this method neither diagnostically useful nor financially practical [1,11,12]. While radiography is the most common, widespread, and cost-effective PMI technique, it is also the weakest in its diagnostic capabilities for PMI, and difficult diagnoses often require additional imaging techniques such as CT or MRI. Nevertheless, this method is recommended as one of the imaging techniques that should be considered the gold standard of perinatal and pediatric PMI.

Fetal Post-mortem Sonography

Fetal post-mortem sonography involves the use of ultrasound sonography to identify the cause of death and unfortunately has not been used or researched extensively in post-mortem settings. However, based on the emerging articles cited in this review, fetal post-mortem sonography is better suited for fetal patients (compared to pediatric imaging), and provides clarifying images of cranial, spinal, and abdominal images when the fetus is too small to be imaged with post-mortem MRI (12-16 weeks), and has high-frequency probes that can be used in close proximity to the target tissue of interest [1]. Granted, post-mortem changes in tissue structure, rigor mortis, and the extended analysis time can complicate further adaptation of the imaging technique [1].

Post-mortem Computed Tomography

**Figure 1:**
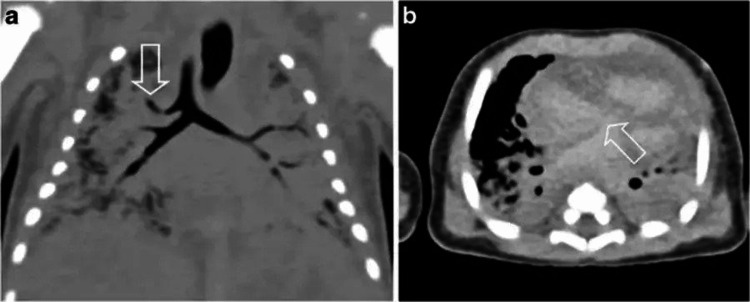
Example of postmortem CT scan in a five-day-old boy: (a) tracheal right upper lobe bronchus is seen via arrow on coronal reconstruction. (b) Atrial septal defect is seen via arrow on axial image.

PMCT is particularly useful for those suffering from internal injuries [13,14]. PMCT is very quick and available; it can also elucidate bone detail, provide a rapid overview of the body interior, and reveal skeletal abnormalities and radiopaque bodies [15]. An example postmortem CT scan of a five-day-old boy to elucidate a cardiovascular cause of death is seen in Figures 1A, 1B; findings were later confirmed by autopsy [16].

While this method may be useful in traumatic deaths (specifically those involving head injuries) and deaths in which foul play is suspected, nearly all studies have procured limited findings suggesting that this method is in any way useful. Articles have shown that it is difficult to determine the cause of death solely through CT scans, insisting that further lab data is required to unequivocally verify diagnoses [1]. This method is far from optimal due to the lack of an intravenous contrast agent, the absence of which limits access to the thoracoabdominal cavity organs, and the inferiority of the soft tissue contrast as a result of reduced abdominal and subcutaneous fat [1]. These cavities are imperative to diagnosis - not only in instances of sudden infant death syndrome (SIDS) but also during gestational development to rule out issues such as respiratory compromise and congenital diaphragmatic hernias. The only way to remedy these shortcomings in late-gestation fetuses is through more invasive direct injection, rendering the imaging redundant.

Studies conducted to test the efficacy of post-mortem CT point to its ineffectiveness. In one Dutch study, post-mortem CT acted as a standard for investigating the cause of death procedures [16]. When observing 54 cases of natural death, the cause of death was identified for only 12.9% of 54 cases, whereas for 74.1% of the 54 cases, PMCT did not add any value to the diagnostic cause of death [16]. In another single-center study, identifying the cause of death for postmortem CT was only possible 40.4% of the time as opposed to 71.4% of the traditional autopsy [17]. Another retrospective study compared the cases of 26 children (0-12 years of age) investigated by autopsy and PMCT whereas, in a large number of the findings, the autopsy proved to be superior to PMCT at detecting organ, soft tissue, and vascular findings while PMCT was superior at detecting bone findings [18]. However, when comparing the diagnostic cases, there was no statistically significant difference in diagnostic accuracy between the two methods [18]. While PMCT is non-invasive and can identify traces of child abuse and causes of death from trauma, the lack of its contrastive ability in soft tissues and vasculature may convince some hospital administrations and jurisdictions do not apply PMCT to patients due to the costs associated with setting up such an apparatus. 

Post-mortem Computed Tomographic Angiography

PMCTA is an examination of an x-ray of blood vessels carried out after the introduction of a radiopaque substance combined with a computerized 3D reconstruction of the vascular system in order to better visualize sources of bleeding for pediatric or perinatal death [15]. PMCTA is the method of choice when determining whether vascular trauma was involved. Vascular pathologies from natural deaths can also be analyzed through this test, enabling the medical examiner to determine the cause of death in perinatal subjects. PMTCA, however, is very time-consuming, needs the preparation of contrast agents and the sample of the study, and requires special equipment: all reasons that can hinder a test that is easily accessible amongst facilities across the country [15].

Post-mortem Magnetic Resonance Imaging

PMMRI involves the use of magnetic fields to image soft tissues and provides excellent contrast for soft tissues [15]. Post-mortem MRIs have been acknowledged for displaying the most promise with the highest diagnostic accuracy out of all the aforementioned imaging techniques among the fetal age group [1]. Its direct diagnoses and ability to guide autopsies to minimize invasive procedures also make it one of the top contenders, even though it requires a longer duration of imaging and necessitates proper training in the fetal anatomy at all stages of development to properly interpret results. The European Society of Pediatric Radiology found that 47/66 of the centers surveyed provided PMI services for children, and 61% of respondents agreed that pediatric PMMRI is an important area that needs to be developed for an accurate post-mortem pediatric diagnosis [12].

**Figure 2:**
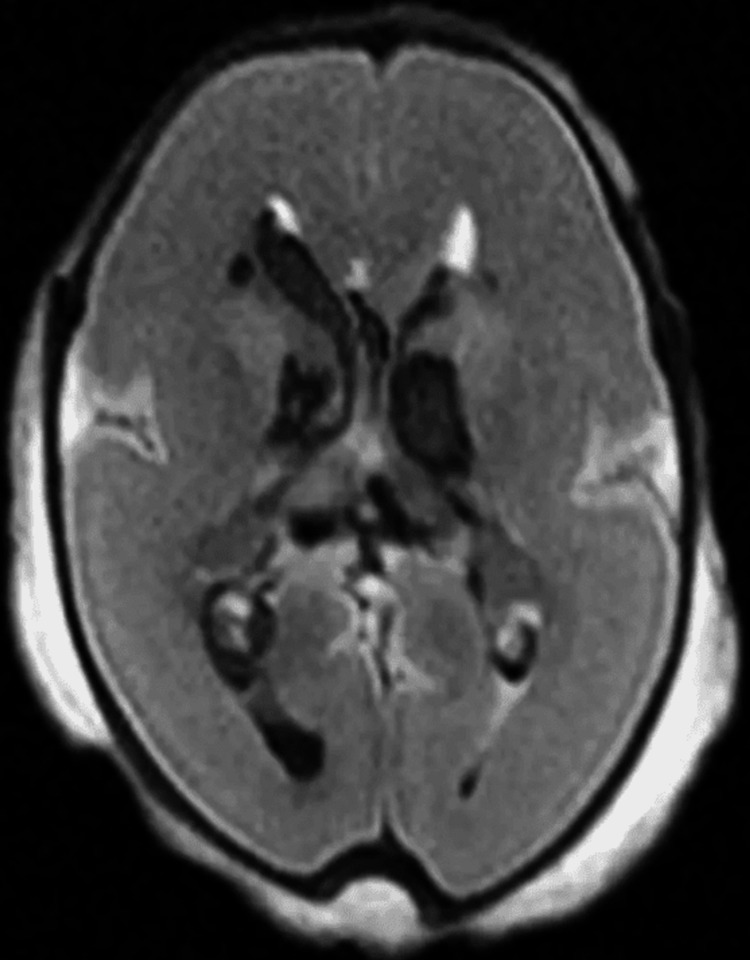
Axial T2-weighted postmortem MRI of brain in a 23-week fetus demonstrates bilateral intraventricular and periventricular hemorrhage.

While PMMRI is extremely complex, it takes time to set up and train technicians and radiologists and requires specific 3D architectural data to be analyzed; it is the most advanced and widespread cross-sectional imaging that can be used for an accurate post-mortem diagnosis especially when loved ones of patients do not want an autopsy [13,19]. A prospective study was also conducted that compared the diagnostic yield of PMCT and PMMRI in which non-invasive PMMRI displayed more accurate diagnoses of major pathologies 24/55 times compared to unenhanced PMCT 18/55 times [1]. One such example is this PMMRI image showing intraventricular and periventricular hemorrhage in a 23-week-old fetus (Figure 2) [1]. It is also important to note that there are cases in which PMMRI is poor such as infection, lung pathology, or effects of size limitations [1].

One innovative clinical study described the accumulation of pleural fluid created as an image from PMMRI as being an important indicator of post-mortem diagnostics for pediatric patients [20]. The same study, however, found no similar relationship between perinatal deaths and stillbirths for unknown reasons [20]. Diffusion-weighted magnetic resonance imaging (DWI) with better models can also be useful in determining apparent brain diffusion coefficient (ADC) and degree of maceration during the gestation and post-mortem interval [21,22]. While PMMRI continues to be on the frontlines for increasing diagnostic capabilities, creating a cost-effective way to construct this apparatus so patients can have more accessible diagnostic tools in medical facilities throughout the nation could be pivotal.

3-Dimensional Surface Scanning

3DSS is a high spatial 3D modeling apparatus that can provide a detailed overview of the body’s surface because of its low maintenance costs and ability to become mobile [15]. 3DSS is useful for immediate trauma assessments or comparison of outside injuries (e.g., cuts, bruises, bites), but no information can be provided regarding internal bodily components. This method, therefore, should be implemented in providing immediate data for pediatric and fetal post-mortem cases that require faster turnaround time or as a precursory, exploratory method.

Using different methods on a single case can allow for the best complex picture and digitalization of data (Table 1). Despite this, techniques are often used independently of one another [15]. Using different methods on a single case can allow for the best complex picture and digitalization of data (Table 1) [1-2, 11-16, 23-32].

**Table 1:** Review of pros and cons of post-mortem prenatal, perinatal, and pediatric imaging modalities

Imaging Modality	Pros	Cons
Post-mortem Radiology	It is useful for identifying long bone fractures and can accurately diagnose skeletal dysplasia in fetus. Quicker turnaround option for hospitals; less imaging resources such as technicians are utilized.	Difficult to assess neonatal deaths due to rigor mortis. Not financially practical and ideal for post-mortem diagnostics.
Post-Mortem Sonography	Provides precise gestational age of subject. and can detect wide range of fetal abnormalities. It may be beneficial in fetal imaging when bodies are too small for other modalities. Useful in identifying congenital abnormalities.	Does not provide useful information for anatomically normal fetuses. Its' efficacy is not widely researched in post-mortem cases.
Post-mortem computed tomography	Could potentially provide sufficient evidence to call for legally investigating cause of death. Quick turnaround time with ease of access in most hospitals. Provides high resolution bone detail and useful in traumatic deaths. Virtual exhumation is possible, has precise identification of foreign bodies, and shows organ volume estimation.	In one Dutch Study, 74.1%showed this method was not of significant value in diagnosing cause of death. Reduced soft tissue contrast. Due to poorer resolution, it is not as beneficial in children when compared to PMMRI.
Post-mortem computed tomography angiography	Good at identifying hemorrhage and skeletal pathology, determination of vascular trauma and health.	Technically difficult. Time-consuming and requires preparation of contrasting agents. Limited ability to detect pathology. Requires special equipment to conduct, usually in tertiary care centers.
Post-mortem magnetic resonance imaging	More than 90%concordance in fetuses and stillbirths when compared to traditional autopsy, and 75% concordance in children. It is useful in determining organ weight or volume estimation, congenital anatomical abnormalities, brain malformations, renal anomalies, congenital heart disease, and skeletal dysplasia. It can also help to identify traumatic soft tissue injuries. Shows complications caused by misplaced intraosseous needles. PMMRI features of feticide can help to differentiate between iatrogenic and physiological processes. Can help determine whether abandoned babies were live births or not; can also estimate approximate time of death. Diffusion-weighted MRI can use natural body decomposition to estimate degree of maceration.	Steep learning curve. Data may be interpreted incorrectly. Weak with finding microscopic changes. Non-diagnostic in approximately 33% of fetuses at less than 24 weeks of gestation. Accuracy decreases at less than 500g body weight. Weak in identifying intestinal and lung pathology; financially costly for many centers.
3-Dimensional Surface Scanning	Can maintain permanent anatomical structure and be useful in teaching/training. Allows for magnification of structure for better examination. Can provide a more palatable method of explaining findings to family and serve as a keepsake to help with grieving process. Beneficial in presenting evidence in legal proceedings.	Technically difficult and expensive to conduct; not widely known or utilized compared to other modalities.

Global availability and protocols of various imaging

Research studies are continually being conducted to identify the optimal imaging technique for post-mortem diagnostics. For example, there is emerging evidence that post-mortem MR may be useful since the body can be left in its plastic cover to analyze [1]. PMCT can be more “jury friendly” than autopsies in providing digital data and accurate transferability of organ volume estimation. The whole-body PMCT angiography testing provides feasible, reliable, and reproducible prenatal congenital cardiac condition diagnostics [15]. In France, the standard approach involving fatalities is through three radiology stations: fluoroscopy, standard radiography, and dental radiography [26]. For a more accurate measure, post-mortem CT can replace one of the tests to replace cross-sectional imaging systems [26]. In the Netherlands, there is a limited role for post-mortem CT and ultrasonography and in most cases, an autopsy will continue to be the method of choice for investigations [3]. While there is a movement toward instructing practitioners to image a post-mortem CT, image quality is reduced due to rigor mortis, and self-reported small sample-sized surveys and studies are still conducted to measure the diagnostic capabilities of the Netherlands [3].

## Conclusions

PMMRI continues to serve as the preferred PMI diagnostic tool in radiology, but it is extremely costly to analyze, set up, and distribute with a lack of accessibility in clinics across the country. Greater adoption of radiological imaging techniques such as PMMRI, PMCT, PMCTA, and 3DSS will, however, allow for more transparent and accurate data for post-mortem diagnostics that can be determined in a non-invasive fashion. Similar to imaging modalities in the clinic space for patients, there are strengths and weaknesses for PMI as well: PMCT is cheaper than PMMRI however it has a poorer resolution for soft tissue etiologies. As noted previously, imaging protocols can be different based on each case’s history or legal framework; for example, in the Netherlands, an autopsy will remain as a cornerstone for post-mortem diagnostics to cover forensic causes (poisoning, non-medically caused deaths of infants). However, inherently as technology advances, so, too, must our standard of care. The healthcare community must continue striving to shift to non-invasive post-mortem prenatal and pediatric diagnostic measures to yield better diagnostic results for both the family and legal bodies in session; an investigation into these imaging modalities would allow for a greater alternative compared to the traditional autopsy procedures.
